# Insights from Coarse-Grained Gō Models for Protein Folding and Dynamics

**DOI:** 10.3390/ijms10030889

**Published:** 2009-03-02

**Authors:** Ronald D. Hills, Charles L. Brooks

**Affiliations:** 1 Department of Molecular Biology and Kellogg School of Science and Technology, The Scripps Research Institute, 10550 N. Torrey Pines Rd. TPC6 La Jolla, CA 92037, USA; 2 Department of Chemistry and Biophysics Program, University of Michigan, 930 N. University Ave, Ann Arbor, MI 48109, USA

**Keywords:** Protein folding, Gō models, coarse-graining, energy landscape, conformational transitions

## Abstract

Exploring the landscape of large scale conformational changes such as protein folding at atomistic detail poses a considerable computational challenge. Coarse-grained representations of the peptide chain have therefore been developed and over the last decade have proved extremely valuable. These include topology-based Gō models, which constitute a smooth and funnel-like approximation to the folding landscape. We review the many variations of the Gō model that have been employed to yield insight into folding mechanisms. Their success has been interpreted as a consequence of the dominant role of the native topology in folding. The role of local contact density in determining protein dynamics is also discussed and is used to explain the ability of Gō-like models to capture sequence effects in folding and elucidate conformational transitions.

## 1. Introduction

Advancing our understanding of biology in the 21^st^ century will involve the structural and dynamical characterization of the countless molecular interactions in the cell. Much can be learned of the structural and dynamical properties of proteins, the workhorses of the cell, from their folding energy landscape. Molecular simulation has emerged as a valuable tool for the detailed exploration of the thermodynamic landscape responsible for guiding the unfolded ensemble through a collection of high energy intermediate structures in order to achieve the proper native structure. We examine recent insights obtained from a simple class of coarse-grained simulation methods for landscape characterization, known as Gō models.

Protein folding is a complex process involving large numbers of degrees of freedom. Therefore, performing traditional molecular dynamics simulations at atomistic detail poses a challenge if one wishes to exhaustively explore the folding landscape of a moderately sized protein with finite computational resources. For this reason coarse-grained representations of the polypeptide chain have been employed in folding simulations. Rather than representing each atom in the protein explicitly groups of atoms can be treated as a single coarse-grained site. In the most common variation, each residue corresponds to a single coarse-grained site placed at the alpha carbon [1–9], although models with multiple sites per residue have been employed [10–14]. Single-site per residue C_α_ models then make up beads on a string, which are held together with harmonic energy terms in the Hamiltonian for the resulting virtual bond and angle vibrational degrees of freedom. Virtual dihedral angles comprising four successive residues can be subjected to potential functions of varying forms, but all share a similar intent of mimicking backbone chirality and Ramachandran conformational preferences [15]. The underpinning of a Gō model, however, is its treatment of nonbonded interactions.

Early coarse-grained models of folding employed the HP recipe, in which hydrophobic residues interact with a constant favorable contact energy (denoted ε) and polar residues either don’t interact or are repulsive. HP models enjoyed limited success as they predicted degenerate global energy minima and failed to identify the unique native state [16]. Current coarse-graining developments endeavor to augment such two-flavor models to contain up to 20 flavors, using distinct physicochemical types to represent each amino acid sidechain or multiple coarse-grained sites per residue for larger sidechains [17–20]. Parameterization of such models to ensure consistency with the all-atom thermodynamic ensemble is a challenging problem. Recent empirical implementations rely on dihedral or hydrogen bond restraints to maintain native secondary structure and are thus of limited use for studying protein conformational changes and folding [21–24]. Two ongoing approaches by the Voth [25] and Scheraga [26] groups offer a promising solution to this problem by employing direct parameterization with all-atom simulation data in order to obtain physically accurate peptide equilibria.

In lieu of a coarse-grained model with physically accurate nonbonded interactions, simplistic Gō models have been applied to the study of folding mechanisms with considerable success. An attractive term of the Lennard-Jones variety [12] is employed for pairwise contacts present in a protein’s native structure, which consists of a shallow energy basin of depth ε that decays to zero for large distances. Residue-residue contacts are typically assigned for nonhydrogen sidechain atoms separated by less than a cutoff distance [12,27], and contacts can also be assigned for backbone hydrogen bonds [2]. All other (non-native) interactions experience a volume-exclusion repulsion. The topology-based approach of favorably distinguishing between native and non-native interactions is named after the early lattice simulation work of Nobuhiro Gō [28–36], and any potential that is biased toward a target structure can be referred to as Gō-like. In Section 2 we discuss the success of Gō models in describing folding. Section 3 follows with recent insights concerning the role of sequence in folding, and, finally, an outlook is presented for the coarse-graining of conformational transitions.

## 2. Role of Native Contacts in Folding

Energy landscape theory posits that proteins have evolved a smooth and funnel-like thermodynamic landscape biased toward the native state, allowing them to fold to a unique stable structure on biological timescales [37–39]. A polypeptide does not have sufficient time to explore all possible conformations in search of the native state; therefore native interactions, whether local or nonlocal [40,41], must play a role in guiding the conformational search in the direction of native structure. As topology-centric Gō models let native interactions drive the folding, their large success has been interpreted as a validation of energy landscape theory.

The reduced degrees of freedom in a coarse-grained representation enable one to exhaustively explore the thermodynamic landscape for folding using standard molecular simulation techniques. In particular, high energy regions of conformational space can be characterized such as the transition state or folding intermediate(s) using either brute-force searching at the folding transition temperature or umbrella sampling with carefully chosen biasing restraints [42]. Gō model simulations can thus be readily compared with experimental phi-value analysis of the thermodynamic effects of point mutations. A phi-value is a qualitative measure of the amount of native-like structure a residue contains in the folding transition state [43]. Phi-values can be predicted for each residue in the protein by averaging over all conformations recorded in the simulation trajectory that correspond to the transition state ensemble. The distribution of native-like secondary and tertiary structure present in the transition state can in part describe the relative order of formation of structural elements, the location of the folding nucleus and hence the overall folding mechanism.

Experimental phi-values have been compared in the literature with predictions from simple C_α_ Gō models for a rapidly growing number of proteins [1–4,8,41,42,44–54]. Qualitative consensus is generally obtained between predictions and experiment for individual residue phi-values as well as the major features of the folding mechanism and folding rate [55]. Non-native interactions have been demonstrated to make some important contributions to the folding transition state and mechanism, and variants of the Gō algorithm have been modified to incorporate nonspecific interactions [4,10,13,56–62]. Notwithstanding, the widespread success of native-only Gō models in reproducing folding behavior as observed in experiment and atomistic simulation [63] suggests that the perturbations of non-native interactions on the structures of folding transition states and intermediates are minor in comparison to the dominating influence of native interactions on the funneled energy landscape [64].

## 3. Sequence Dependence of Folding

Over the last decade the folding community has emphasized the native topology as the main determinant of folding behavior [39,65]. The topology-centric paradigm has resulted from the rise of energy landscape theory, the prevalence and success of Gō modeling studies, and the notion of contact order. Defined as the average sequence separation between contacting residues in the native state, contact order is inversely correlated with experimental folding rates, allowing kinetic predictions to be made based on the local or nonlocal nature of complex backbone topologies. Small helical proteins are known to fold faster than large β-sheet proteins due to the more local nature of their contacts [66,67]. Nonetheless, it is a protein’s sequence that determines the particular topology it chooses to adopt. Single residue mutations can significantly alter the kinetics of folding and the stability of the native state. Amyloid disease-causing mutations lead to partially unfolded forms that self-associate to form pathogenic oligomers and aggregates [68,69]. The precise role of sequence in folding remains to be understood.

Methods have been employed for extending simple Gō models to examine sequence effects in folding. Rather than weighting native contacts equally by assigning the same interaction energy ε to all contacts, energetic heterogeneity can be introduced by scaling residue-residue interactions according to physically motivated criteria. Native interactions have been optimized in Src-SH3, CI2, protein G and S6 using thermodynamic perturbation data from mutation experiments [64,70]. Clementi and coworkers optimized native and non-native contact energies in Src-SH3 using an iterative procedure to maximize the energy gap between the folded and unfolded states [57]. Dokholyan and coworkers scaled contact energies based on atomistic simulations with the CHARMM force field that identified a small set of critical residues in SOD1 folding [71]. Karanicolas and Brooks [2] employed a general set of residue-residue interactions using the tabulated potentials of Miyazawa and Jernigan (MJ) derived from statistical analysis of contacts in the Protein Data Bank [72]. Native contact energies have also been weighted by crystallographic B-factors [73] and hydrogen exchange protection factors [74] with success. One last method that can be considered to constitute a ‘flavored’ Gō model is the all-atom Gō approach [75–85]. The protein is represented at atomic resolution, and nonbonded atom-atom pairs within a cutoff distance are assigned a favorable contact potential. As larger sidechains are more likely to be assigned a higher number of atom-atom contacts, they are apt to interact more favorably than smaller sidechains.

Energetic heterogeneity adds ruggedness to the folding landscape and, more importantly, allows for the investigation of sequence effects in, for example, the folding of proteins with identical topology. The MJ-flavored model of Karanicolas and Brooks was used to investigate symmetry breaking in protein L and protein G, two proteins that share a common topology consisting of N- and C-terminal β-hairpins and a central α-helix. The balance between enthalpic interactions and chain entropy was observed to lead to N-terminally nucleated folding in protein L and C-terminal nucleation in protein G, consistent with experimental findings [2]. The same model was recently employed to compare the folding mechanisms for three members of the common flavodoxin fold [42,86,87]. CheY, NtrC and Spo0F each consist of five βα-repeats arranged into a central β-sheet and surrounding α-helices. Consistent with experimental findings for CheY [88,89], all three proteins were observed to obey an N-terminal nucleation mechanism in simulations due to abundant contacts in the N-terminus. A larger number of contacts in the C-terminal subdomain of Spo0F were observed to lead to some differences in its folding mechanism, however.

While Clementi *et al* had previously observed N-terminal nucleation in simulations of CheY with a simple Gō model [1], a more detailed analysis was undertaken by Hills *et al*. to compare the role of sequence in CheY homologs [42,86,87]. The free energy landscape was characterized as a function of the formation of the various secondary and tertiary structural elements within the 120-residue proteins in order to map out the relative order of structure formation. Though more rigorous computational methods exist for determining the folding transition state ensemble [90,91], using structural coordinates to monitor folding progress in simulations has proven valuable in the elucidation of complex folding mechanisms in large proteins when multiple reaction coordinates are needed to explain folding behavior [49,92,93]. Transition path sampling methods were recently employed to analyze the reaction coordinate for the 20-residue Trp-cage mini-protein [94]. Two structural coordinates were needed to best describe its parallel folding pathways of helix and tertiary contact formation. The fraction of native contacts formed, denoted *Q*, is a progress variable of considerable utility [92,95–97]. Folding in CheY and homologs was shown to be well described by native contact formation in the N- and C-terminal subdomains [42,86,87].

**Figure 1.**
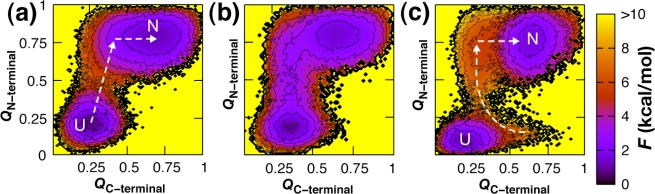
N-terminally nucleated folding landscapes for structural homologs CheY (a), NtrC (b) and Spo0F (c). The equilibrium free energy was computed as a function of the fraction of native contacts formed within the N- (*Q*_N-terminal_) and C-terminal (*Q*_C-terminal_) subdomains and is shown at the transition temperature at which the folded and unfolded states are equally populated [42,87].

While CheY and NtrC contain an alanine-rich C-terminal cavity that is dynamic in the unphosphorylated state and important for function, in Spo0F this region is filled in with bulkier residues such as isoleucine and exhibits rigidity, having lost the C-terminal conformational allostery of its evolutionary homologs [98–101]. The enhanced stability and van der Waals contacts in this region were shown to lead to competitive frustration in the folding landscape (Figure 1). Whereas in CheY and NtrC the N-terminal subdomain formed easily and rapidly nucleated C-terminal folding, in Spo0F C-terminal contacts were seen to form ahead of and temporarily preclude N-terminal folding.

Simulation results are in accord with experimental analysis of the three homologs; the kinetics of folding were found to be slowest in Spo0F, suggesting an interesting relationship between function and folding mechanism in the CheY family [87,88]. Comparison of the folding mechanisms for the CheY family, T4 lysozyme and interleukin-1β suggests that subdomain competition is a general property of multimodule proteins [42,47,102]. It is interesting to note that the contact energies assigned by the flavored Gō model were uniformly distributed throughout the three CheY-like proteins. The local contact density, defined as the number of native contacts per residue, could alone explain folding behavior, implying that an unflavored Gō model could also have possibly captured the main sequence effects in the three homologs. This notion is supported by a recent comparison study of results obtained from flavored and unflavored Gō models [103]. For β-sheet proteins in which tertiary contacts are well distributed throughout the protein, introducing energetic heterogeneity was observed to negligibly impact the folding mechanism; for α-helical bundles held together by only a handful of tertiary interactions, interaction heterogeneity was observed to alter folding.

## 4. Conclusions and Outlook

The role of contact density in dictating protein folding and dynamics is increasingly being recognized. Contact density has been used to explain B-factors [104] and downhill folding [54]. A comprehensive survey of experimental and simulation data for homodimers revealed that high interface hydrophobicity and a large ratio of interfacial to monomeric contacts are indicative of an obligate two-state association mechanism, in which dimerization precedes monomer folding [50,51]. Two-state dimers commonly have an intertwined or pseudo domain-swapped interface. Cooperativity in hydrophobic packing was shown to be sufficient for nucleating amyloid aggregation [105]. Residue fluctuations and the location of folding nuclei have been explained by treating proteins as small-world networks consisting of a regular lattice with a fraction of the edges between vertices replaced with new random connections [106–112]. Clusters of the branched aliphatic sidechains isoleucine, leucine and valine have been identified as particularly important in stability and folding [87,88,113,114]. In order to predict hydrogen exchange protection factors, a combinatorial algorithm was used to generate an ensemble of unfolded conformational states and the thermodynamic probability of each state was estimated using considerations of chain entropy and solvent exposed surface area [115]. A survey of large two-subdomain proteins revealed that native contact density can distinguish stable, nucleating subdomains from dynamic subdomains involved in function [86]. Correlations between contact density and folding/dynamics help to explain the success of coarse-grained models in biomolecular simulation. Nowhere is this success more surprising than in the case of analytical Ising models of folding that consider chain entropy and native contact enthalpy while neglecting the three-dimensional spatial representation of the peptide chain [116–120].

The role of native contacts in protein dynamics is best appreciated in the context of elastic network normal mode analysis (EN-NMA). To calculate vibrational modes in large systems proteins can be represented as an elastic network of C_α_-beads in which residues within a cutoff distance experience harmonic interactions. The lowest frequency normal modes of vibration are then easily computed for the elastic network model. Often, a single or small subset of low frequency vibrations has been shown to reproduce the large amplitude structural rearrangements associated with biological function [19,121–123]. EN-NMA has successfully reproduced the functional dynamics of systems as diverse as adenylate kinase, myosin, RNA polymerase, the ribosome, GroEL-GroES and viral capsids [124,125]. The success of EN-NMA in capturing functional motions has been interpreted as a consequence of nature’s exploitation of shape, topology and packing in the evolution of protein conformational dynamics [124,126–128].

A disadvantage of EN-NMA is that ‘closed’ to ‘open’ conformational transitions cannot be observed as they would involve the breaking of harmonic bonds in the network. Alternative methods exist for computing the minimum free energy path between two conformational states based on interpolation between the two reference elastic networks for each state. Optimization algorithms can be employed to find between the two global minima either a single minimum energy pathway [129] or an ensemble of low energy paths reflecting the ruggedness of protein landscapes [130]. The double-well approach has recently been employed successfully with Gō models, in which native interaction potentials were included for residue contacts present in either of two reference conformations [131–133]. The so-called dual, or switching, Gō model was used to study conformational transitions in myosin, Arc repressor and F1-ATPase.

The pervasive role of contact density can explain the robustness of the many varieties of Gō models that have been used to study folding. The results obtained with a Gō model are sensitive to the model parameters and various energy functional forms used [5,6,12,134–136]. A common means to safeguard against this is to compare folding results for multiple proteins obtained using the same model in light of available experimental data. Simulation results can also be influenced by the experimental structure from which the Gō model is derived, particularly the method used in its solution [137,138]. Note that NtrC exhibits a lower folding barrier than CheY and Spo0F in Figure 1, a result of having fewer native contacts overall and therefore a smoother landscape. The sparser native contacts in NtrC can be attributed to its being based on an NMR solution structure as opposed to the crystal structures for CheY and Spo0F [87]. It is also not uncommon for Gō models to be employed with native dihedral and/or angle restraints in addition to the typical nonbonded native interactions in order to make folding more cooperative [1,139]. Introducing a small desolvation barrier in the nonbonded native interaction potential has been employed as an alternate means of ensuring folding cooperativity [2,12,61,140].

Sulkowska and Cieplak recently compared a total of 62 different variations of the Gō potential in reproducing the experimental mechanical unfolding data of 28 proteins [12]. Several of the common functional forms employed for the native contact potential along with both uniform and heterogeneous energy scales were able to accurately reproduce the maximum unfolding force in stretching simulations (*R*^2^ ≈ 0.84). Most of the other models investigated came in a close second tier with *R*^2^ > 0.70 for the correlation between predicted and experimental unfolding forces. Whitford *et al*. have compared results obtained from C_α_ and all-atom Gō models of three proteins [85]. While the C_α_ models neglected sidechain packing considerations, the correct global folding mechanisms were still obtained. Such studies suggest that the many variations of Gō models that have been employed to date are more alike than they are dissimilar.

Current non-Gō coarse-grained models depend on some form of secondary structure restraints to ensure native state stability [21–24]. Improving the physical accuracy of these non-Gō interactions will be a necessary step in removing biasing potentials from coarse-grained models so that large scale conformational transitions can be more readily studied. Native structure restraints aside, empirical coarse-graining techniques can often be considered Gō-like for their very nature of neglecting detailed atomistic interactions whilst favoring the essential interactions of the system [141–144]. In lieu of a physically accurate and transferable non-Gō coarse-grained model for proteins, Gō-like models will continue to find application in the study of protein folding and dynamics. The mechanical unfolding of titin, ubiquitin, bacteriorhodopsin and knotted transcarbamylase has recently been simulated using Gō potentials [76,145–149]. A Gō modeling survey of 7,510 structures in the Protein Data Bank was used to further delineate the topological determinants of mechanical stability [150]. A 297-residue outer membrane protease has been studied using a hybrid atomistic/coarse-grained approach in which the active site was represented with an atomistic force field and the protein scaffold was described using a Gō model [151–153]. Symmetrized Gō potentials have been employed for domain swapping and amyloid aggregation in which intermonomer residue contacts are treated identical to intramonomer native contacts [13,14,154]. Gō model simulations have been performed to explore the influence of macromolecular crowding agents [155,156] and confinement [62] on folding. Electrostatic interactions were incorporated between a Gō model of a transcription factor and coarse-grained DNA, which caused the protein to visit partially unfolded forms and suggested a fly casting mechanism of target recognition [157]. Lastly, in light of the connections with contact density, the link between function and folding mechanism is becoming a topic of considerable investigation [86,158–164].
